# Soft robotic exosuit augmented high intensity gait training on stroke survivors: a pilot study

**DOI:** 10.1186/s12984-022-01034-2

**Published:** 2022-06-03

**Authors:** Sung Yul Shin, Kristen Hohl, Matt Giffhorn, Louis N. Awad, Conor J. Walsh, Arun Jayaraman

**Affiliations:** 1 Max Nader Lab for Rehabilitation Technologies and Outcomes Research, Shirley Ryan AbilityLab, 355 E Erie St., Chicago, IL 60611 USA; 2 Department of Physical Medicine and Rehabilitation, Northwestern University, 710 N Lake Shore Dr, Chicago, IL 60611 USA; 3 College of Health and Rehabilitation Sciences: Sargent College, Boston University, Boston, USA; 4 Harvard John A. Paulson School of Engineering and Applied Sciences, Harvard University, Cambridge, USA; 5 Wyss Institute for Biologically Inspired Engineering, Harvard University, Cambridge, USA

**Keywords:** Exosuit, Soft robotics, High intensity gait training, Clinical outcomes, Gait quality, Stroke

## Abstract

**Background:**

Stroke is a leading cause of serious gait impairments and restoring walking ability is a major goal of physical therapy interventions. Soft robotic exosuits are portable, lightweight, and unobtrusive assistive devices designed to improve the mobility of post-stroke individuals through facilitation of more natural paretic limb function during walking training. However, it is unknown whether long-term gait training using soft robotic exosuits will clinically impact gait function and quality of movement post-stroke.

**Objective:**

The objective of this pilot study was to examine the therapeutic effects of soft robotic exosuit-augmented gait training on clinical and biomechanical gait outcomes in chronic post-stroke individuals.

**Methods:**

Five post-stroke individuals received high intensity gait training augmented with a soft robotic exosuit, delivered in 18 sessions over 6–8 weeks. Performance based clinical outcomes and biomechanical gait quality parameters were measured at baseline, midpoint, and completion.

**Results:**

Clinically meaningful improvements were observed in walking speed ($p$ < 0.05) and endurance ($p$ < 0.01) together with other traditional gait related outcomes. The gait quality measures including hip ($p$ < 0.01) and knee ($p$ < 0.05) flexion/extension exhibited an increase in range of motion in a symmetric manner ($p$ < 0.05). We also observed an increase in bilateral ankle angular velocities ($p$ < 0.05), suggesting biomechanical improvements in walking function.

**Conclusions:**

The results in this study offer preliminary evidence that a soft robotic exosuit can be a useful tool to augment high intensity gait training in a clinical setting. This study justifies more expanded research on soft exosuit technology with a larger post-stroke population for more reliable generalization.

*Trial registration* This study is registered with ClinicalTrials.gov (ID: NCT04251091)

## Introduction

Stroke is a leading cause of serious long-term disability [1], leaving the majority of those who survive with pervasive gait deficits such as reduced walking speed, decreased endurance, and atypical gait patterns [2]. Improving walking ability is a high-rated priority for individuals following a stroke, and a major goal of physical therapy interventions [3].

One of the emerging therapeutic regimens to improve functional outcomes after stroke involves high intensity gait training with the focus on higher cardiovascular intensities [4]. Indeed, previous studies suggested that the amount and intensity of stepping training are related to gains in walking speed and endurance [5]. This strategy currently is primarily focused on improving functional locomotor capacity, with less quality of movements [6]. The potential neglect of control in quality of movements during the training may lead to persistence of gait impairments such as spatiotemporal asymmetries and gait compensations [7, 8], ultimately resulting in a metabolically inefficient gait [9] and an increased risk of falling [10].

Soft robotic exosuits are portable, lightweight, and unobtrusive assistive devices made from garment-like functional textiles, cable-based actuators and wearable sensors to improve the mobility of post-stroke individuals through facilitation of more normal paretic limb function during walking [11–13]. Previous studies have demonstrated strong evidence of the immediate gait restorative effects using soft robotic exosuits on post-stroke individuals. For instance, prior foundational studies reported improvements in the mechanics and energetics of post-stroke hemiparetic walking as well as clinical outcomes (i.e., walking speed and endurance) with assistance provided to the paretic ankle in plantarflexion and dorsiflexion by a soft robotic exosuit [12–15]. A recent Robotic Exosuit Augmented Locomotion (REAL) trial conducted a high-intensity, task-specific, and progressively challenging walking training protocol with soft robotic assistance and demonstrated improvements both in clinical and biomechanical outcomes after 5 days of training on a single post-stroke individual [16]. Another recent multi-site clinical study tested a commercially- and clinically-available soft exosuit on post-stroke participants and reported increased maximum walking speed after five sessions of training [17]. These studies showed initial evidence that a single-session, acute restorative effects can be further leveraged to improvements when the soft exosuit is used with gait training in a short-term period. However, at present, it is uncertain how the longer duration (matching outpatient therapy models) of high intensity gait training augmented with soft exosuit will impact traditional clinical measures and biomechanical quality of gait movements post-stroke.

The objective of this pilot intervention study was to examine the rehabilitative effects of soft robotic exosuit-augmented gait training on clinical and biomechanical outcomes of gait in post-stroke individuals. We conducted 18 training sessions of high intensity gait training using a soft exosuit on five individuals in the chronic phase of post-stroke recovery. The main clinical outcomes of the intervention were improving walking speed and endurance as they are the primary walking goals of the physical therapy intervention after stroke [18, 19]. The secondary outcomes were the gait quality measures including spatiotemporal characteristics and joint kinematics to evaluate changes in gait impairments throughout the intervention. We hypothesized that this combined training would improve the traditional clinical outcomes together with biomechanical gait quality measures due to the synergistic effect of training on both intensity and quality of movements.

## Methods

### Participants

Participants were recruited between December 2019 and January 2021 from Shirley Ryan AbilityLab (formerly Rehabilitation Institute of Chicago). Inclusion criteria for the trial were as follows: age 18–80 years old, stroke event occurred at least 6 months ago, observable gait deficits, able to walk without the support of another person for at least 2 min (without an assistive device or orthotic support), passive ankle dorsiflexion range of motion to neutral with the knee extended (i.e., able to achieve an angle of 90° between the shank and foot), and physician approval. Exclusion criteria were as follows: score of > 1 on question 1b and > 0 on question 1c on the NIH Stroke Scale [20], inability to communicate with investigators, neglect or hemianopia, unexplained dizziness in the last 6 months, pressure ulcers or skin wounds located at human-device interface sites, known urethane allergies, history of significant Peripheral Artery Disease (PAD), unresolved Deep Vein Thrombosis (DVT), pregnancy and other comorbidities that prevent full participation in the research.

A convenience sample of five community dwelling individuals post-stroke participated in this pilot study and all participants completed the intervention program. Demographic information for these participants is reported in Table 1. All participants were in chronic phase of stroke, with an average latency of 2.7 ± 1.92 years.

**Table 1:** Demographics and clinical characteristics of participants

	P1	P2	P3	P4	P5
Age (years)	37	38	54	51	52
Sex	Female	Male	Male	Male	Male
BMI (kg/m^2^)	23.4	28.2	27.1	34.0	24.4
Stroke subtype	Hemorrhagic	Ischemic	Ischemic	Ischemic	Ischemic
Hemiparesis	Right	Right	Right	Right	Left
Stroke latency (years)	3.2	5.8	1.4	2.5	0.8
Lower limb orthosis users	Ankle brace	AFO	None	None	AFO
Assistive device users	None	None	None	None	Straight cane
Comorbidities	None	Other cardiovascular disease	Hypertension	Other cardiovascular disease	Hypertension
Optimal timing parameter	Late	Early	Late	Late	Late

*BMI* body mass index, *AFO* ankle foot orthosis

### Soft exosuit device

**Fig. 1:**
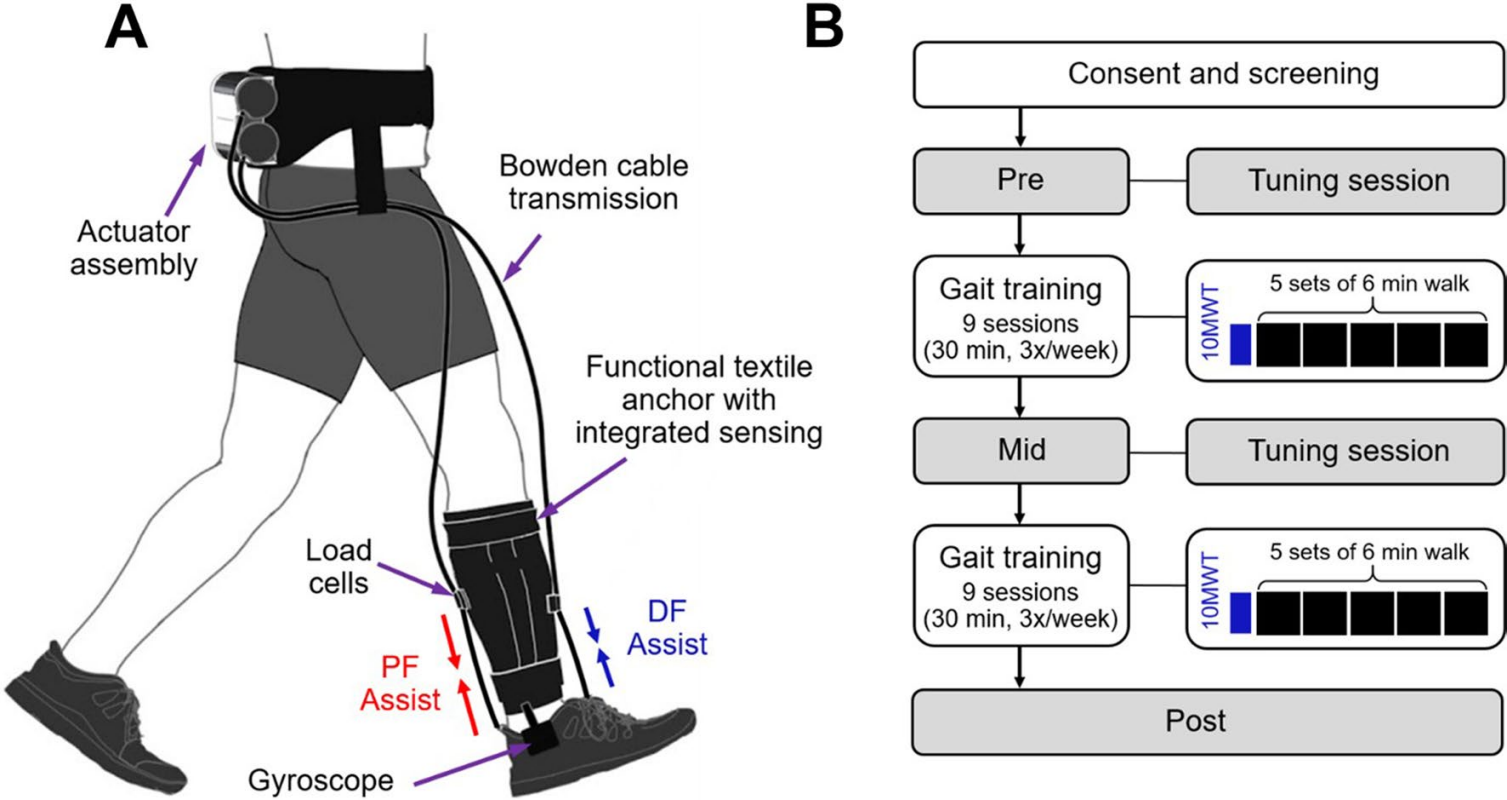
**A** The ReWalk ReStore™ (ReWalk Robotics, Israel) soft robotic exosuit designed to assist paretic ankle dorsiflexion and plantarflexion of individuals with post-stroke. **B** Workflow of intervention, including assessment and training of high intensity gait training augmented with soft robotic exosuit. Gait training sessions consist of initial 10MWT to determine baseline self-selected speed followed by five sets of 6-min walking on treadmill or overground. Pre: before training; Mid: after 9 training sessions; Post: after 18 training sessions; 10MWT: 10-Meter Walk Test

The ReWalk ReStore™ (ReWalk Robotics, Israel) is a powered, lightweight, and commercially-available soft robotic exosuit intended for use in stroke rehabilitation of individuals with lower limb disability [17]. The soft exosuit was designed to interface with the paretic limb of people post-stroke and has components worn proximally at the waist and distally on the paretic shank and shoe (see Fig. 1A) [13, 21]. It provides dynamic plantarflexion and dorsiflexion assistance during walking intended to restore paretic limb function resulting in improved foot clearance, increased propulsion symmetry, reduced gait compensations, and reduced metabolic burden of hemiparetic gait [12–14].

The overall weight of the exosuit is approximately 5 kg, with most of the weight located proximally in the actuation pack worn at the waist. The components worn at the waist consist of a mechanical actuator, battery, and the functional textile anchor used to securely attach the components to the user. The components worn at the shank and shoe consist of a sensor assembly containing load cell and gyroscope, a functional textile anchor worn around the shank and a shoe insole that integrate the sensors and transmit power generated by the mechanical actuator to the targeted ankle joints via Bowden cables. The Bowden cables located anterior and posterior to the ankle assist with dorsiflexion during the paretic swing phase and plantarflexion during late stance phase, respectively. The bilateral gait events were detected in real time using the gyroscopes worn on each shoe and used to control the dorsiflexion and plantarflexion assistances.

The soft exosuit has three preset modes that include assist (provide the pre-configured dorsiflexion and plantarflexion assistance), slack (no assistance), and brace (maintain in a fixed, configurable ankle position) [17]. The device was set to assist mode for all participants throughout the study with optimal assistance timing parameter determined during the tuning session described in the following section.

### Interventions

The intervention timeline of the participants is illustrated in Fig. 1B. Intervention consisted of therapist-guided gait training for 30 min per session, ~ 3 times per week for 6–8 weeks for a total 18 training sessions. The 18 sessions for the study duration were chosen to mimic Medicare reimbursement guidelines for standard outpatient stroke rehabilitation. All sessions were administered by a licensed physical therapist.

At the initial visit (Day 0), consent and determination of study eligibility was performed with standard clinical measures administration. Additionally, fitting and familiarization with the soft exosuit for a few minutes on a treadmill and overground were completed at this visit. In the following session (Day 1, tuning session), the baseline assessment including 2-min walk test on a treadmill and overground was conducted without wearing the device (Pre). GAITRite instrumented walkway (CIR Systems, Inc., NJ) and inertial measurement unit (IMU) motion tracking sensors (Xsens, Enschede, The Netherlands) were used to capture baseline spatial and temporal parameters as well as joint kinematics during walking. Exosuit parameter tuning was then performed by systematically assessing three plantarflexion timing conditions to identify optimal timing: early (20%), middle (50%) and late timing (90% of stance phase). The optimal timing was selected based on the objective information including spatial and temporal gait symmetry from GAITRite data, subjective information including therapist’s observation, and patient’s preference. If GAITRite data was similar between conditions, we relied on patient preference and therapist observation to make a decision. The selected optimal timing for each subject is reported in Table 1. Once the optimal timing was determined (i.e., early, middle, or late timing), the identical timing parameter was used for the first 9 training sessions. Additionally, the amount of dorsiflexion was tuned to provide adequate toe clearance through swing phase.

After the tuning session, subjects completed 9 training sessions (Day 2–10). At the beginning of every training session, the 10-Meter Walk Test (10MWT) was performed without the device to measure the baseline self-selected gait speed. The target speed of the training was determined as 115% of the measured self-selected speed that date [16]. High intensity gait training is defined based on the higher cardiovascular intensities (e.g., 70–85% heart rate maximum) during walking or stepping practice [22]. While our approach was to encourage patient’s engagement based on target speed, the high intensity was achieved by the nature of increased walking speed from the patient’s self-selected speed. The high intensity was confirmed during the gait training by monitoring real-time heart rate data obtained by a heart rate monitor (Polar OH1, Polar Electro, Kempele, Finland). Subjects completed 30 min of gait training broken down into five sets of 6-min on a treadmill or overground with the device. The goal of each bout was to achieve the predetermined target speed. If the subject was able to maintain the target speed for two consecutive bouts on the treadmill, they were transitioned to overground walking. The patients were allowed to have rest breaks between sets as needed. After the first 9 training sessions, subjects completed a midpoint assessment session (Mid) with another tuning session. None of the subjects switched their assistance timing from the previous tuning session demonstrating stronger preference towards familiar tuning parameters. Subjects then completed 9 additional training visits (Day 12–20) followed by the post assessment session (Post).

### Assessments

#### Clinical outcomes

Participants’ clinical outcomes were assessed at three time points without wearing the soft exosuit or orthotics: before training (Pre), after 9 training sessions (Mid), and after 18 training sessions (Post). All assessments were performed by a licensed physical therapist. Each assessment included performance-based outcome measures including the 10-Meter Walk Test (10MWT), 6-min walk test (6MWT), Functional Gait Assessment (FGA), Timed-Up-and-Go (TUG), lower extremity subscale of the Fugl-Meyer Assessment (LE-Motor-FM), 2-min walk test (2MWT) on overground and treadmill.

#### Gait quality measures

To assess gait quality measures, spatiotemporal characteristics and joint kinematics were captured by GAITRite instrumented walkway and IMU motion tracking sensors (Xsens) during 2MWT on a treadmill, respectively. Spatiotemporal parameters included step length and step time, defined as the linear distance between right and left feet, and duration of each step, respectively. For the joint kinematic parameters, the range of motion (RoM) from sagittal plane joint variables, including hip and knee flexion/extension and ankle dorsiflexion/plantarflexion were analyzed. Additionally, we observed ankle angular velocity obtained from the IMU motion tracking sensors as the assistance of paretic ankle plantarflexion and dorsiflexion during walking is the major function of the soft exosuit. Top panel of Fig. 3B illustrates the example of ankle angular velocity profiles across a single gait cycle at Pre, Mid and Post from a representative subject (Patient 5). We used area under the curve to quantify the measure of ankle angular velocity at each time point.

All gait quality parameters involved unaffected and affected sides, enabling symmetry analysis. The symmetry index metric was used to evaluate the gait symmetry given by

1 $${SI}_{n}=\frac{{US}_{n}-{AS}_{n}}{0.5({US}_{n}+{AS}_{n})}\times 100 [\%]$$

where ${US}_{n}$ and ${AS}_{n}$ are the *n*th gait parameter of the unaffected and affected side, respectively, and $n$ can be the aforementioned spatiotemporal and joint kinematic parameters [6, 8]. The value is always between − 200 and 200%, and a positive (or negative) value indicates ${US}_{n}>{AS}_{n}$ (or vice versa). Note that the symmetry index ${SI}_{n}=0\%$ when the gait parameter between unaffected and affected sides is in perfect symmetry (i.e., ${US}_{n}={AS}_{n}$).

### Statistical and minimal important difference analyses

Statistical analyses were performed using R version 3.6.1 (2019 R foundation for statistical computing). Significant level was set to $\alpha =0.05$ unless otherwise noted. A generalized linear mixed effects models were used to examine the percentage changes in each clinical outcome and gait quality measure over time, including time points (Mid and Post) as a fixed effect and subject as a random effect. Due to small sample size, a residual analysis was performed to check for normality. Post hoc tests determined whether changes from baseline were significantly different from 0 at Mid, or Post time points, and significant level for these tests were adjusted using Tukey honestly significant difference [23].

To clinically evaluate the effects of the intervention, the primary outcome measures of 10MWT and 6MWT were compared with the minimal clinically important differences (MCID). Difference in walking speed at Post from Pre was compared with 0.14 m/s MCID of 10MWT [24]. In addition, difference in walking endurance at Post from Pre was compared with 34.4 m MCID of 6MWT [25]. These numbers are from the acute-stroke population, currently there are no published MCID values for clinical outcomes specific to chronic stroke [26]. As the secondary outcome measures, the FGA and TUG scores were also compared with their minimal detectable changes (MDC). Difference in FGA score at Post from Pre was compared with 4.2 points MDC of FGA [27]. In addition, difference in TUG score at Post from Pre was compared with 2.9 s MDC of TUG [28].

## Results

### Clinical assessments

**Fig. 2:**
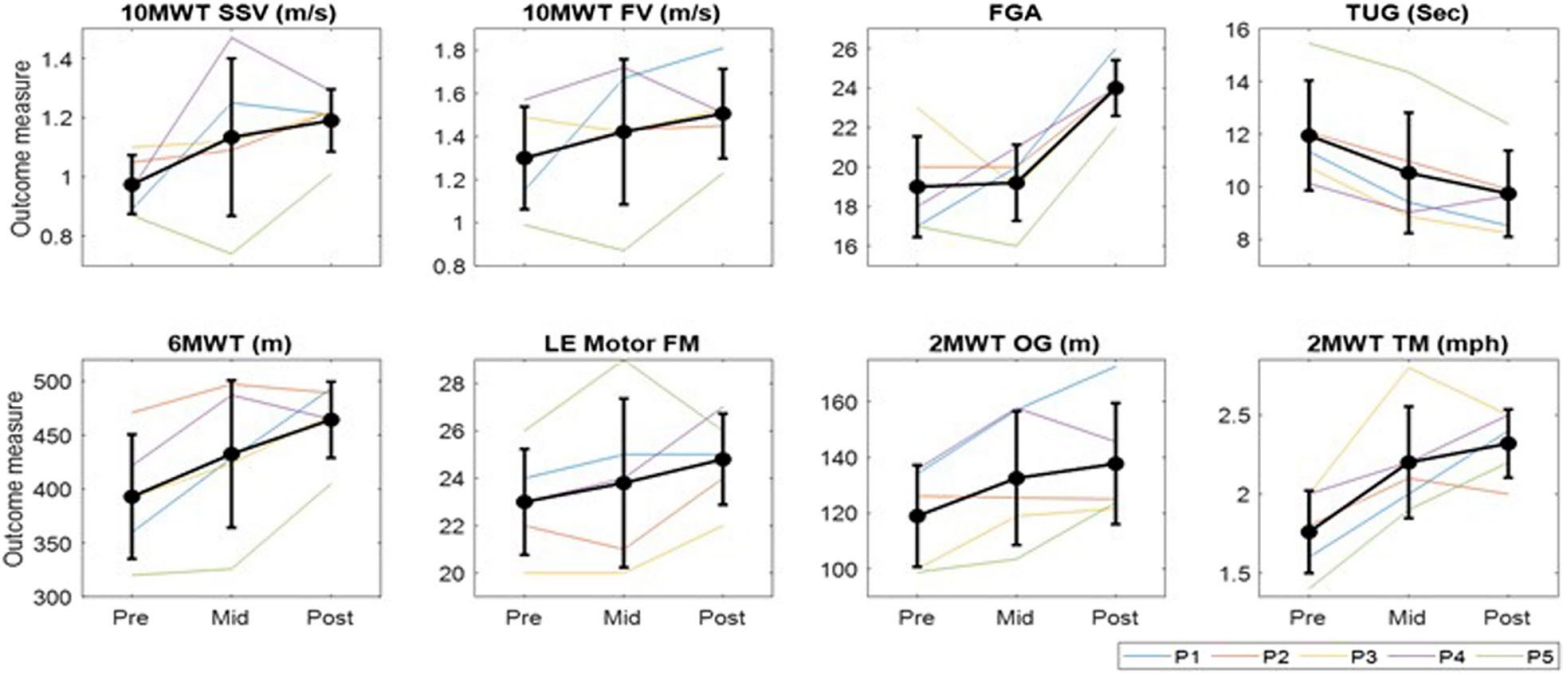
Clinical outcome measures before training (Pre), after 9 training sessions (Mid), and after 18 training sessions (Post) time points. 10MWT SSV: 10-Meter Walk Test for self-selected walking velocity; 10MWT FV: 10-Meter Walk Test for fast walking velocity; FGA: functional gait assessment; TUG: Timed-Up-and-Go; 6MWT: 6-min walk test; LE Motor FM: lower extremity subscale of the Fugl-Meyer Assessment; 2MWT OG: 2-min walk test overground, 2MWT TM: 2-min walk test treadmill

All five subjects completed gait training and assessment sessions with no adverse events. We first observed the clinical assessment scores over time points (i.e., Pre, Mid, and Post) to qualitatively inspect overall changes in participants’ functional recovery as a result of the intervention. Figure 2 illustrates the progression of clinical outcome scores at Pre, Mid, and Post assessment sessions (see also Table 2). Overall, we observed improving trends in all clinical outcome measures over the three time points.

**Table 2:** Changes in clinical outcome scores over time points and those scores relative to before training

Clinical outcomes	Mean absolute score (mean ± SD)			Mean relative score (mean ± SD)	
	Pre	Mid	Post	Mid–Pre	Post–Pre
10MWT-SSV, m/s	0.97 ± 0.10	1.13 ± 0.27	1.19 ± 0.11	0.16 ± 0.22	0.26 ± 0.10
10MWT-FV, m/s	1.30 ± 0.24	1.42 ± 0.34	1.51 ± 0.21	0.12 ± 0.25	0.21 ± 0.28
6MWT, m	392.8 ± 57.7	432.5 ± 68.3	464.3 ± 35.3	39.7 ± 27.0	71.5 ± 43.9
FGA, points	19.0 ± 2.5	19.2 ± 1.9	24.0 ± 1.4	0.2 ± 2.9	5.0 ± 2.9
TUG, sec	11.9 ± 2.1	10.5 ± 2.3	9.7 ± 1.6	− 1.4 ± 0.44	− 2.2 ± 1.0
LE-Motor-FM, points	23.0 ± 2.2	23.8 ± 3.6	24.8 ± 1.9	0.8 ± 1.5	1.8 ± 1.5
2MWT-OG, m	118.9 ± 18.2	132.5 ± 24.0	137.7 ± 21.8	13.6 ± 10.8	18.8 ± 15.1
2MWT-TM, mph	1.76 ± 0.26	2.20 ± 0.35	2.32 ± 0.22	0.44 ± 0.23	0.56 ± 0.25

10MWT-SSV: 10-Meter Walk Test for self-selected velocity; 10MWT-FV: 10-Meter Walk Test for fastest walking velocity; 6MWT: 6-min walk test, FGA: Functional Gait Assessment; TUG: Timed-Up-and-Go Test; LE-Motor-FM: lower extremity subscale of the Fugl-Meyer Assessment; 2MWT-OG: 2-min walk test for overground walking; 2MWT-TM: 2-min walk test for treadmill walking; Pre: before training; Mid: after 9 training sessions; Post: after 18 training sessions; SD: standard deviation

For the quantitative analysis, we used linear mixed effects models to examine the percentage changes in clinical outcome scores over time points. Table 3 reports the average percentage change in scores at Mid and Post assessments from Pre after the intervention. Residual analysis determined that data is likely from a normal distribution. The results indicated that there was a main effect of time points on all outcome measures (all $p$ < 0.05) except for fast walking speed ($p$ = 0.07). Post hoc analyses revealed that there were significant improvements at Post from Pre in self-selected walking speed as measured by the 10MWT (22.7 ± 11.6%, $p$ < 0.05), walking endurance as measured by the 6MWT (19.5 ± 13.2%, $p$ < 0.01), FGA (28.0 ± 17.9%, $p$ < 0.01), TUG (− 18.1 ± 8.0%, $p$ < 0.01), LE-Motor-FM (8.13 ± 6.56%, $p$ < 0.05), and 2 min overground (16.43 ± 12.65%, $p$ < 0.01) and treadmill (33.65 ± 19.22%, $p$ < 0.01) walking.

**Table 3:** Model results and post-hoc tests of percent changes in clinical outcomes relative to before training

	Linear mixed effect model		Post hoc test (mean ± SD)	
	Slope [95% CI]	$p$-value	Mid–Pre	Post–Pre
Self-selected walking speed, 10MWT-SSV	11.35 [1.12, 21.58]	< 0.05	16.85 ± 28.63	22.70 ± 11.59*
Fast walking speed, 10MWT-FV	9.20 [− 1.10, 19.51]	0.07	9.59 ± 22.05	18.41 ± 24.20
Walking endurance, 6MWT	9.75 [4.33, 15.18]	< 0.01	10.05 ± 7.16	19.51 ± 13.15**
Functional gait assessment, FGA	14.00 [5.42, 22.58]	< 0.01	2.21 ± 15.02	28.01 ± 17.86**
Timed-Up-and-Go, TUG	− 9.06 [− 12.57, − 5.55]	< 0.01	− 12.29 ± 4.65	− 18.12 ± 8.01**
LE-Motor-FM	4.06 [0.53, 7.60]	< 0.05	3.10 ± 5.96	8.13 ± 6.56*
2-min overground walking	8.22 [2.80, 13.63]	< 0.01	11.32 ± 8.64	16.43 ± 12.65**
2-min treadmill walking	16.82 [8.02, 25.63]	< 0.01	25.48 ± 12.58**	33.65 ± 19.22**

10MWT-SSV: 10-Meter Walk Test for self-selected velocity; 10MWT-FV: 10-Meter Walk Test for fastest walking velocity; 6MWT: 6-min walk test; FGA: Functional Gait Assessment; TUG: Timed-Up-and-Go Test; LE-Motor-FM: lower extremity subscale of the Fugl-Meyer Assessment; CI: confidence interval; Pre: before training; Mid: after 9 training sessions; Post: after 18 training sessions; SD: standard deviation

*p < 0.05; **p < 0.01

Then the primary outcome measures (i.e., 10MWT and 6MWT) were compared with the MCIDs to clinically examine the effect of the intervention. The average improvements of 10MWT-SSV and 10MWT-FV at Post from Pre were 0.22 ± 0.1 and 0.21 ± 0.28 m/s, respectively, greater than established MCID (0.14 m/s) of the 10MWT. Among five participants, four participants’ 10MWT-SSV and three participants’ 10MWT-FV surpassed MCID of the 10MWT. The average improvement of 6MWT at Post from Pre was 71.5 ± 43.9 m, with four participants surpassing MCID (34.4 m) of the 6MWT. The secondary outcome measures including FGA and TUG scores were also compared with their established MDCs. The average improvements of FGA score at Post from Pre was 5.0 ± 2.92 points, with three participants surpassing MDC (4.2 points) of the FGA. However, the average improvements (i.e., decrease) of TUG score at Post from Pre was 2.2 ± 1.0 s not exceeding the MDC (2.9 s) of TUG. Among five participants, only one participant surpassed MDC of the TUG.

### Gait quality measures

**Fig. 3:**
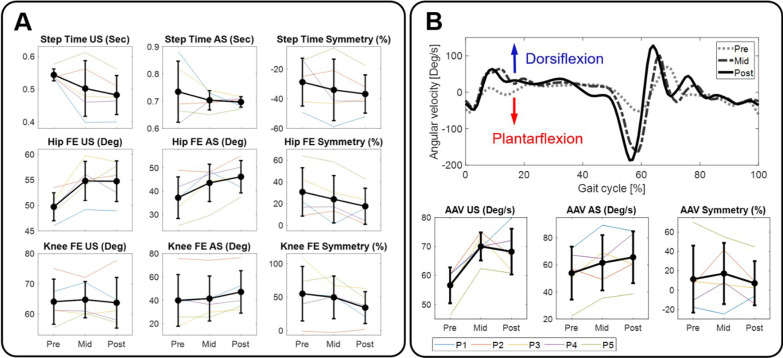
**A** Selected gait quality measures with significance before training (Pre), after 9 training sessions (Mid), and after 18 training sessions (Post) time points. **B** Example of ankle angular velocity profiles across a single gait cycle from a representative subject (top panel) and changes in ankle angular velocity at Pre, Mid and Post time points (bottom panel). *US* unaffected side, *AS* affected side, *FE* flexion/extension, *AAV* ankle angular velocity

We observed the changes in gait quality measures including spatiotemporal and sagittal plane joint kinematic parameters over time points (i.e., Pre, Mid, and Post) to investigate how the intervention affected the quality of movements during walking. Figure 3A and B illustrate qualitative visualization of (A) the selected gait parameters that revealed significant changes (i.e., step time, hip and knee flexion/extensions) and (B) ankle angular velocity over time points (see also Table 4).

**Table 4:** Changes in gait quality measures over time points and relative to before training

Clinical outcomes	Mean absolute score (mean ± SD)			Mean relative score (mean ± SD)	
	Pre	Mid	Post	Mid–Pre	Post–Pre
Spatiotemporal characteristics					
Step time US, s	0.54 ± 0.02	0.50 ± 0.08	0.48 ± 0.06	− 0.04 ± $0.07$	− 0.06 ± 0.05
Step time AS, s	0.73 ± 0.11	0.70 ± 0.04	0.70 ± 0.02	− 0.03 ± 0.09	− 0.04 ± 0.11
Step length US, m	58.4 ± 7.9	61.1 ± 12.3	63.9 ± 7.8	2.6 ± 6.3	5.4 ± 5.8
Step length AS, m	65.7 ± 9.5	68.3 ± 13.7	71.8 ± 8.1	2.6 ± 8.0	6.2 ± 5.0
Joint range of motion					
Hip Flex/Ex US, °	49.70 ± 2.73	54.77 ± 3.83	54.70 ± 3.98	5.06 ± 2.86	5.00 ± 3.07
Hip Flex/Ex AS, °	37.19 ± 8.91	43.53 ± 7.98	46.16 ± 6.94	6.33 ± 5.08	8.97 ± 3.61
Knee Flex/Ex US, °	64.09 ± 7.46	64.76 ± 6.00	63.73 ± 8.37	0.66 ± 3.18	− 0.36 ± 2.62
Knee Flex/Ex AS, °	39.85 ± 22.18	41.44 ± 19.31	47.09 ± 18.21	1.60 ± 6.19	7.25 ± 7.08
Ankle Dorsi/Plantar US, °	35.27 ± 6.30	37.37 ± 3.04	37.64 ± 4.24	2.10 ± 7.24	2.37 ± 3.99
Ankle Dorsi/Plantar AS, °	31.77 ± 14.50	31.31 ± 9.98	34.46 ± 10.62	− 0.46 ± 6.81	2.69 ± 7.45
Hip Flex/Ex US, °	49.70 ± 2.73	54.77 ± 3.83	54.70 ± 3.98	5.06 ± 2.86	5.00 ± 3.07
Ankle kinematics					
Ankle angular velocity US, °/s	65.63 ± 6.18	70.06 ± 4.82	68.25 ± 7.91	13.43 ± 3.81	11.63 ± 5.72
Ankle angular velocity AS, °/s	53.93 ± 19.58	61.57 ± 20.47	65.68 ± 19.14	7.64 ± 12.04	11.75 ± 5.18

US: unaffected side; AS: affected side; Flex/Ex: flexion/extension; Dorsi/Plantar: dorsiflexion/plantarflexion; Pre: before training; Mid: after 9 training sessions; Post: after 18 training sessions; SD: standard deviation

Table 5 shows quantitative analysis of the average percentage change in gait quality measures including spatiotemporal parameters, joint RoM and ankle angular velocity at Mid and Post from Pre. Residual analysis determined that data is likely from a normal distribution. In spatiotemporal characteristics, we observed significant change in step time of unaffected side ($p$ < 0.05) and increasing trend in step length of both sides, although not statistically significant ($p$ = 0.07 and $p$ = 0.08 for unaffected and affected side, respectively). In joint RoM, the results indicated that there were significant increases in hip flexion/extension of both sides ($p$ < 0.05 and $p$ < 0.01 unaffected and affected sides, respectively), and knee flexion/extension of affected side ($p$ < 0.05). While our data did not indicate any significant changes in RoM of the ankle dorsiflexion/plantarflexion ($p$ = 0.27 and $p$ = 0.19 for unaffected and affected side, respectively), the ankle angular velocity revealed significant increase on both unaffected and affected sides (both $p$ < 0.05). Post hoc analyses indicated that there were significant decrease in step time of unaffected side at Post (− 11.45 ± 8.86%, $p$ < 0.05) from Pre, significant increase in RoM of hip flexion/extension of both sides at all other time points (10.09 ± 6.21% and 26.80 ± 16.31% increase at unaffected and affected side, respectively at Post, both $p$ < 0.01) from Pre, significant increase in knee flexion/extension of affected side (29.16 ± 32.25%, $p$ < 0.05) at Post from Pre, and significant increase in ankle angular velocity of both sides (20.91 ± 10.71% and 28.21 ± 26.22% increase at unaffected, $p$ < 0.01, and affected sides $p$ < 0.05, respectively at Post) at Post from Pre.

**Table 5:** Model results and post-hoc tests of percent changes in gait quality relative to before training

Gait parameters	Linear mixed effect model		Post hoc test (mean ± SD)	
	Slope [95% CI]	$p$-value	Mid–Pre	Post–Pre
Spatiotemporal characteristics				
Step time US	− 5.72 [− 10.47, − 0.98]	< 0.05	− 7.86 ± 13.40	− 11.45 ± 8.86*
Step time AS	− 6.98 [− 1.70, 3.59]	0.49	− 2.92 ± 11.75	− 3.39 ± 14.61
Step length US	4.89 [− 0.38, 10.15]	0.07	3.82 ± 11.27	9.77 ± 9.67
Step length AS	4.99 [− 0.68, 10.66]	0.08	3.55 ± 12.96	9.98 ± 8.50
Joint range of motion				
Hip Flex/Ex US	5.04 [1.48, 8.61]	< 0.05	10.22 ± 5.62**	10.09 ± 6.21**
Hip Flex/Ex AS	13.40 [5.33, 21.47]	< 0.01	18.68 ± 14.21*	26.80 ± 16.31**
Knee Flex/Ex US	− 0.31 [− 2.95, 2.33]	0.80	1.33 ± 5.16	− 0.61 ± 4.01
Knee Flex/Ex AS	14.58 [1.71, 27.45]	< 0.05	11.51 ± 30.38	29.16 ± 32.25*
Ankle Dorsi/Plantar US	4.09 [− 3.80, 11.99]	0.27	8.95 ± 22.14	8.19 ± 12.41
Ankle Dorsi/Plantar AS	10.71 [− 6.19, 27.61]	0.19	11.57 ± 42.93	21.41 ± 40.17
Ankle kinematics				
Ankle angular velocity, US	10.45 [2.96, 17.94]	< 0.05	24.37 ± 8.95**	20.91 ± 10.71**
Ankle angular velocity, AS	14.11 [1.74, 26.47]	< 0.05	20.31 ± 29.75	28.21 ± 26.22*
Gait symmetry				
Step time	− 4.00 [− 9.57, 1.57]	0.14	− 5.24 ± 14.23	− 8.01 ± 11.59
Step length	− 0.14 [− 2.55, 2.27]	0.90	0.43 ± 5.52	− 0.28 ± 6.32
Hip Flex/Ex	− 6.58 [− 11.29, − 1.87]	< 0.05	− 6.70 ± 9.92	− 13.16 ± 6.64**
Knee Flex/Ex	− 10.41 [− 19.91, − 0.91]	< 0.05	− 5.41 ± 20.98	− 20.82 ± 20.58*
Ankle Dorsi/Plantar	− 3.60 [− 14.35, 7.15]	0.47	1.53 ± 24.53	− 7.20 ± 18.90
Ankle angular velocity	− 2.12 [− 11.51, 7.27]	0.62	5.84 ± 20.89	− 4.24 ± 13.78

US: unaffected side; AS: affected side; Flex/Ex: flexion/extension; Dorsi/Plantar: dorsiflexion/plantarflexion; CI: confidence interval; Pre: before training; Mid: after 9 training sessions; Post: after 18 training sessions; SD: standard deviation

*p < 0.05; **p < 0.01

We then examined the changes in symmetry of these gait parameters over time points. Table 5 reports the changes in symmetry index of gait parameters over time points based on linear mixed effect model (see also Fig. 3). No significant changes were observed in symmetry of spatiotemporal parameters ($p$ = 0.14 and $p$ = 0.90 for step time and step length, respectively). In joint kinematics, however, we observed that there were significant changes in symmetry of hip and knee flexion/extension (both $p$ < 0.05). Post hoc analyses revealed that there were significant improvements in symmetry of hip (− 13.16 ± 6.64%, $p$ < 0.01) and knee (− 20.82 ± 20.58%, $p$ < 0.05) flexion/extension at Post from Pre.

## Discussion

The primary goal of this pilot study was to explore the therapeutic potential of high intensity gait training augmented with soft robotic exosuit on clinical and biomechanical outcomes of gait in individuals in the chronic phase of stroke recovery. The main findings were as follows: first, the primary outcome measures including self-selected walking speed (e.g., 10MWT) and walking endurance (e.g., 6MWT) revealed significant improvements greater than MCIDs after the intervention. Second, the gait quality measures including hip and knee flexion/extension exhibited an increase in range of motion in a symmetric manner, suggesting that the restoration of the walking function was achieved also by reducing gait impairments. To our knowledge, this study was the first long-term intervention study that translated soft exosuit technology as a part of clinic-based rehabilitation program. The results in this study support that soft exosuit can potentially be a useful tool to provide therapeutic benefit on both functional and biomechanical outcomes in a clinical rehabilitation setting.

After intervention, improvements in walking speed (10MWT) and endurance (6MWT) were observed above the MCIDs [24, 25] together with other traditional gait related clinical outcomes including Functional Gait Assessment, lower extremity subscale of the Fugl-Meyer Assessment, and 2-min walk test. Particularly, we observed that the walking speed in the 10MWT increased by 0.22 m/s at Post assessment session after 18 sessions of 30-min gait training. This is comparable with a previous study of traditional high intensity stepping training without any device, consisting of 36 ($\pm$ 5.8), 1-h sessions that revealed average 0.23 m/s increase in 10MWT at Post session [5]. Although the baseline scores were different (i.e., starting from a lower baseline of 0.44 m/s in the traditional high intensity stepping training study), training with the soft exosuit yielded similar performance gains after less than half the therapy time/dose. In addition, we observed continuous improvements in most of the clinical outcomes until the Post assessment session. This suggests that there is an additional room for improvements and this could motivate a follow-up study with a longer training period.

The results from this study are also comparable with a previous study that demonstrated improvements in clinical outcomes after the intervention with Honda’s Stride Management Assist (SMA) exoskeleton, which provides assistance at the hip (consisted of therapist-guided gait training at 45 min per session, total 18 sessions over 6–8 weeks) [29]. In the present study, the walking speed in the 10MWT and distance walked in the 6MWT increased by 22.7% (0.22 m/s) and 19.5% (71.5 m) at Post assessment session, respectively. Comparatively, the SMA intervention revealed walking speed in 10MWT and distance walked in the 6MWT increased by 33.5% (0.24 m/s) and 46.0% (116.9 m), respectively. The greater improvements in these clinical scores in SMA intervention could be due to the ceiling effect of our subjects, since our baseline scores (0.97 m/s and 392.8 m for 10MWT and 6MWT, respectively) were considerably higher than the SMA intervention group (approximately 0.70 m/s and 260 m for 10MWT and 6MWT, respectively). Note that the inclusion criteria of the SMA intervention involved initial walking speed 0.4–0.8 m/s (limited community ambulators). Despite this ceiling effect, the present study (1.19 m/s and 464.3 m for 10MWT and 6MWT, respectively) revealed significantly greater scores at post assessment session than the SMA intervention (approximately 0.95 m/s and 375 m for 10MWT and 6MWT, respectively). However, since the results of the present study were based on a small sample size, more expanded research with a larger population is needed for a more reasonable comparison.

A secondary objective of this study was to examine the additional effects of the intervention on spatiotemporal and joint kinematic parameters. Notably, we observed positive changes in gait quality measures including significant increase in hip and knee flexion/extension ($p$ < 0.01 for affected hip flexion/extension and $p$ < 0.05 for unaffected hip and affected knee flexion/extension) with improved symmetry over time points (both $p$ < 0.05 for hip and knee flexion/extension symmetries). These results agree with a previous soft robotic exosuit study that demonstrated increase in forward propulsion with reduction in propulsion asymmetry [13, 16], given that there exists a significant association between trailing limb angle (i.e., related to hip and knee joint motion) and gait propulsion [30]. Overall, given that the improvements in clinical outcomes were observed together with gait quality measures within shorter therapy time, we argue that this may be a synergetic effect of combined high intensity gait training augmented with soft robotic exosuit.

With the assistance provided to the paretic ankle joint from the soft exosuit during the training, we observed significant increase in ankle angular velocity on both sides (unaffected, $p$ < 0.01, and affected side $p$ < 0.05) at post assessment session. This is consistent with a previous study that showed an increase in ankle joint angular velocity with increased walking speed as our participants walked faster after the intervention [31]. However, the range of motion at ankle joint did not exhibit any significant changes over time points. This could be related to the guidance hypothesis that provision of too much assistance during training with the exosuit may have caused the participants to develop an overreliance on the exosuit, resulting in non-adaptation/learning in ankle motion during walking without the device [32, 33]. For this study, the exosuit was used in active assistance mode for all training. However, it is possible to program an intermittent (i.e. where the cables go slack to enable true mechanical transparency) or progressively reduced assistance paradigm for some portion of a gait training protocol to ensure that the wearer is challenged to use their volitional effort at the ankle [16]. This may help to maximize the recovery by allowing patients to train more with their own intention instead of relying too much on the assistance from the soft robotic exosuit.

The primary limitations of this proof-of-concept study included a small sample size of five subjects with a restricted range of impairment level—all participants were community ambulators with gait speed > 0.8 m/s (average speed: 0.97 ± 0.1 m/s) [34], and lack of follow-up assessment sessions and a control group. These limit the generalizability to a larger population of stroke and remain an open question on persistency of the rehabilitative effects. In addition, it is difficult to separate the contributions of the soft exosuit and high intensity gait training due to the lack of control group in this study. For instance, some previous studies of traditional high intensity stepping training revealed changes in gait quality measures (e.g., kinematics) although none of them reported improvements in kinematic symmetry [35, 36]. Nevertheless, our data still showed a potential that implementing soft robotic exosuit training into a clinical intervention (i.e., high intensity gait training) can provide valuable therapeutic effects on both functional ability as well as quality of movements. We expect this study will justify more expanded research with a larger post-stroke population to establish more detailed effectiveness and reliable generalization.

While the kinematic gait quality measures provide more global picture of gait recovery post-stroke, capturing joint kinematics may be relatively more sensitive to errors than measuring conventional clinical outcomes (e.g., speed, endurance, etc.) [37]. Typical drawbacks of IMUs, magnetic distortion and improper calibration of hemiparetic walking, may have influenced our results. For instance, our data did not indicate any significant trend in trailing limb angle, possibly because the IMUs were not accurate enough to detect absolute joint movements due to the misaligned posture during calibration. To mitigate these issues, we used an IMU motion capture system specifically designed for capturing kinematics [38–40] and limited our gait quality measures to range of motion of sagittal plane joint kinematics. Another limitation of this work included lack of neuromuscular analysis of participants’ walking using electromyography (EMG). However, we speculate that the soft exosuit intervention would have positively influenced the neuromuscular properties of our participants given that there is a strong relationship between gait quality measures and neuromuscular control parameters [41]. Future work incorporating additional measurements including three dimensional joint kinematics and EMG analysis would provide more comprehensive characterization of therapeutic effect on gait recovery with the intervention.

## Conclusion

The purpose of this preliminary study was to investigate the therapeutic effect of high intensity gait training augmented with a soft robotic exosuit on clinical and biomechanical outcomes of gait in chronic stroke individuals. We observed that participants improved walking speed and endurance together with other traditional gait related clinical outcomes (e.g., Functional gait assessment, Timed-Up-and-Go, etc.). In addition, the gait quality measures including hip and knee flexion/extension showed increased range of motion and improved symmetry, suggesting walking function was improved together with gait quality. The results in this study offer preliminary evidence that the soft exosuit can be a useful tool to provide therapeutic value. These promising initial results justify further research of the soft exosuit intervention in a larger clinical study.

## Data Availability

De-identified data are available from the authors upon reasonable request.

## Abbreviations

10MWT: 10-Meter Walk Test
2MWT: 2-Min walk test
6MWT: 6-Min walk test
AFO: Ankle foot orthosis
AS: Affected side
BMI: Body mass index
Dorsi/Plantar: Dorsiflexion/plantarflexion
DVT: Deep vein thrombosis
EMG: Electromyography
FGA: Functional Gait Assessment
Flex/Ex: Flexion/extension
FV: Fast walking speed
IMU: Inertial measurement unit
MCID: Minimal clinically important difference
MDC: Minimal detectable change
ME-Motor-FM: Fugl-Meyer Assessment
Mid: After 9 training sessions
OG: Overground walking
PAD: Peripheral artery disease
Post: After 18 training sessions
Pre: Before training
REAL: Robotic Exosuit Augmented Locomotion
RoM: Range of motion
SD: Standard deviation
SMA: Stride Management Assist exoskeleton
SSV: Self-selected walking speed
TM: Treadmill walking
TUG: Timed-Up-and-Go Test
US: Unaffected side
