# Correction to: Soft robotic exosuit augmented high intensity gait training on stroke survivors: a pilot study

**DOI:** 10.1186/s12984-022-01080-w

**Published:** 2022-09-19

**Authors:** Sung Yul Shin, Kristen Hohl, Matt Giffhorn, Louis N. Awad, Conor J. Walsh, Arun Jayaraman

**Affiliations:** 1 Max Nader Lab for Rehabilitation Technologies and Outcomes Research, Shirley Ryan AbilityLab, 355 E Erie St., Chicago, IL 60611 USA; 2 Department of Physical Medicine and Rehabilitation, Northwestern University, 710 N Lake Shore Dr., Chicago, IL 60611 USA; 3 College of Health and Rehabilitation Sciences: Sargent College, Boston University, Boston, USA; 4 Harvard John A. Paulson School of Engineering and Applied Sciences, Harvard University, Cambridge, USA; 5 Wyss Institute for Biologically Inspired Engineering, Harvard University, Cambridge, USA

## Correction to: Journal of NeuroEngineering and Rehabilitation (2022) 19:51 https://doi.org/10.1186/s12984-022-01034-2

Following the publication of the original article [1], the text “*Patents describing the exosuit components documented in this article have been filed with the U.S. Patent Office. They are licensed by ReWalk Robotics and C.J.W. is an inventor. CJW is a paid consultant for ReWalk Robotics*” has been included under the Competing interests.

The original article has been corrected.
